# Carbon Fiber 3D Printing: Technologies and Performance—A Brief Review

**DOI:** 10.3390/ma16237311

**Published:** 2023-11-24

**Authors:** Gabriele Marabello, Chiara Borsellino, Guido Di Bella

**Affiliations:** Department of Engineering, University of Messina, 98166 Messina, Italy; chiara.borsellino@unime.it (C.B.); guido.dibella@unime.it (G.D.B.)

**Keywords:** additive manufacturing (AM), carbon fiber 3D printing, versatility, continuous fiber printing, short fiber printing, polymer matrix

## Abstract

Additive manufacturing is evolving in the direction of carbon fiber 3D printing, a technology that combines the versatility of three-dimensional printing with the exceptional properties of carbon fiber. This work aims to provide a brief review of the main methodologies used in carbon fiber 3D printing, focusing particularly on the two most widespread types: continuous fiber printing and short fiber printing. In the context of continuous fiber printing, the process of embedding a continuous carbon fiber into a polymer matrix will be examined, resulting in the achievement of high-performance lightweight structural components. On the other hand, short fiber printing involves the use of short carbon fibers mixed in turn with polymeric materials, with the advantage of having greater ease of processing and obtaining highly performing components with large-scale economic investments that are lower in cost than additive manufacturing using continuous fiber printing. Furthermore, this work will conduct an evaluation of the mechanical properties of products printed using both technologies, focusing on key aspects, such as strength, stiffness, weight, and resistance to mechanical stress. The specific advantages and challenges associated with each printing technique will also be analyzed.

## 1. Introduction

Three-dimensional (3D) printing, also known as additive manufacturing (AM) or rapid prototyping (RP), is an innovative production technology that enables the fabrication of solid objects layer by layer using three-dimensional model data [1]. Unlike traditional subtractive manufacturing methods, 3D printing allows on-demand production of finished products from computer-aided design (CAD) models. This technology originated in the 1980s and has evolved as a versatile technology for accomplishing complex structures using a wide range of materials [2], including carbon-based materials, metals [3], biological materials, ceramics, and even food. 3D printing has multiple applications in various sectors, such as construction, aerospace, medicine, and many others [4], both for academic research and industrial production. Carbonaceous materials, thanks to their excellent chemical properties, versatile nanostructure, and special mechanical [5,6], electrochemical, thermal, and electrical properties, have been widely used in 3D printing. In recent years, there has been a growing interest in using 3D printing to manufacture complex systems containing carbon fibers, whether short or long. Various 3D printable carbonaceous materials have been developed, including carbon-filled composites, carbon precursors, and other variants. These materials offer significant opportunities for achieving advanced functional systems. Despite current challenges, such as optimizing the properties of printed materials, increasing carbon content, and achieving high-carbon structures, progress in 3D printing of carbon continues to push the boundaries of additive manufacturing. Research and development in this field are ongoing to fully harness the potential of 3D printing of carbon and explore new emerging applications.

## 2. Results

Carbon-based materials are widely utilized in 3D printing due to their remarkable chemical stability, versatile nanostructure, exceptional mechanical, electrochemical, thermal, and electrical properties. Over the past years, significant progress has been made in 3D printing, particularly in the field of energy applications, enabling the fabrication of intricate systems. Extensive research studies have been focused on the 3D printing of carbon-based materials, including carbon fibers [7], carbon nanotubes, graphene, and graphene oxide. Nevertheless, the printing of pure carbon materials is still undergoing development, primarily due to technical obstacles associated with the fusion, sintering, or polymerization of pure carbon. The exploration of 3D printing for glassy and porous carbon materials has received relatively less attention. Challenges include the optimization of properties and porosity of printed materials, augmenting the carbon content, and achieving structures with a high carbon composition. Recognizing the diverse array of carbonaceous materials and the myriad 3D printing techniques is significant to fully exploit the immense potential of this technology [8].

In recent years, several types of 3D printing processes aimed at producing carbon structures have been studied and developed. The main technological developments and the methodologies they use will be analyzed. The most widely developed technology involves the mechanical reinforcement of polymers through the addition of carbon fibers. Different results have been achieved based on the morphology of the fibers and the mechanical process used to bond these fibers to the polymer.

Fiber-reinforced polymer matrix composites offer exceptional directional mechanical properties, and the integration of these characteristics with the capability of 3D printing has led to numerous innovative advancements in research. In addition to typical composite guiding factors, such as fiber orientation, fiber volume fraction, and stacking sequence, it has been demonstrated that printing parameters including infill density, infill pattern, nozzle speed, layer thickness, build orientation, and nozzle and bed temperatures influence the mechanical properties of printed composites [9,10].

The main additive manufacturing technologies through which it is possible to obtain carbon structures will be analyzed, including both the most innovative techniques and the most commonly used and most easily accessible ones linked to fused deposition modeling technology, i.e., all those technologies in which carbon fiber is impregnated in polymeric materials and then deposited in layers through a nozzle. Technologies that use continuous carbon fiber and those that use short fibers will be analyzed more specifically. The mechanical properties of the finished component that has been produced using these technologies will be discussed.

### 2.1. 3D-Printed Continuous Carbon Fiber-Reinforced Composites

Considering the effectiveness demonstrated in various studies regarding the integration of carbon fiber for enhancing the mechanical performance of polymers commonly used in additive manufacturing, there has been a growing fascination with producing 3D-printed components utilizing continuous carbon fiber. In the field of additive manufacturing, the integration of continuous fibers into the plastic resin of 3D printed models represents a recent and promising innovation with the potential to define the next generation of composite materials. This technology has demonstrated a significant contribution to enhancing the mechanical properties of composites, including tensile strength, flexural strength, compression resistance, and impact resistance. Its widespread acceptance stems from its ability to generate intricate designs with a reduced number of process steps and the freedom to fabricate reinforcements as needed. Moreover, it has led to substantial improvements, often several times greater, compared to unreinforced composites, paving the way for the potential use of continuous fiber fabrication (CFF) composites in high-load-bearing applications. However, despite the advantages, the commercial application of this technology remains somewhat limited. This is primarily due to the anisotropic behavior of CFF composites, poor interfacial bonding, high porosity, and the restricted range of materials (matrix and reinforcement) available for customizing composite properties according to specific needs. Additionally, CFF composites exhibit lower mechanical performance, as evidenced by meso- and microstructural studies, in comparison to traditionally prepared composites. Other significant hurdles include challenges related to time, cost, and scalability. It is, therefore, possible to state that research into CFF composites is still in an exploratory phase, with a predominant focus on varying printing parameters, processes, and reinforcement configurations, rather than the discovery of new applications or suitable alternatives to currently used materials. Despite the challenges, the industrial potential of this technology remains considerable, but further research and development are necessary to overcome current limitations and fully unlock the opportunities offered by 3D printing of continuous fiber-reinforced composites [11,12,13,14].

**Figure 1:**
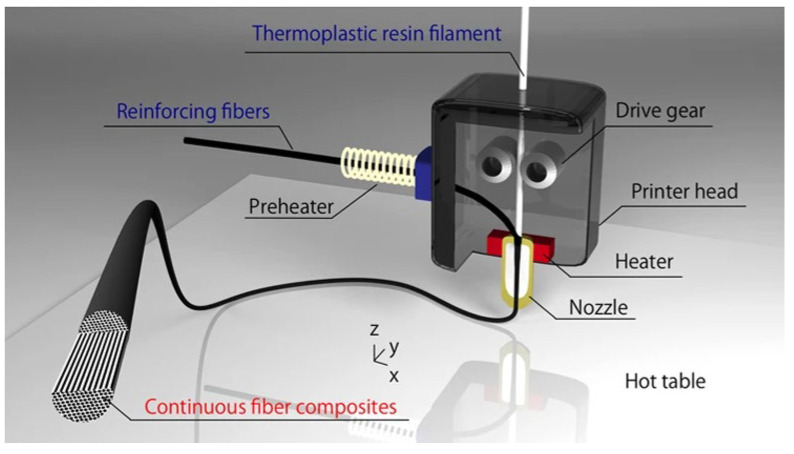
Schematic of the 3D printer head used to produce continuous FRTPs using an in-nozzle ([16], reproduced under a CC BY 4.0 license).

Li et al., 2016 [15] explores the use of 3D printing technology to manufacture continuous carbon fiber-reinforced polylactic acid (PLA) composites. It involves a specialized nozzle design to ensure uniform mixing of carbon fibers and PLA resin during printing and addresses the weak bond between carbon fibers and PLA through fiber preprocessing. Experimental results show substantial improvements in mechanical and thermal properties. Tensile and flexural strengths of preprocessed carbon fiber-reinforced composites are 13.8% and 164% higher than untreated samples, respectively. The storage modulus also surpasses pure PLA and untreated samples, with increases of 166% and 351%, respectively. Scanning electron microscope (SEM) analyses confirm enhanced fiber-resin bonding. This rapid 3D printing technology has promising applications in creating complex, high-performance components, especially in aerospace. Other studies have developed similar methodologies; specifically, Matsuzaki et al., 2016 [16] have developed an innovative technique for 3D printing of continuous fiber-reinforced thermoplastic composites, based on fused deposition modeling (see Figure 1).

**Figure 2:**
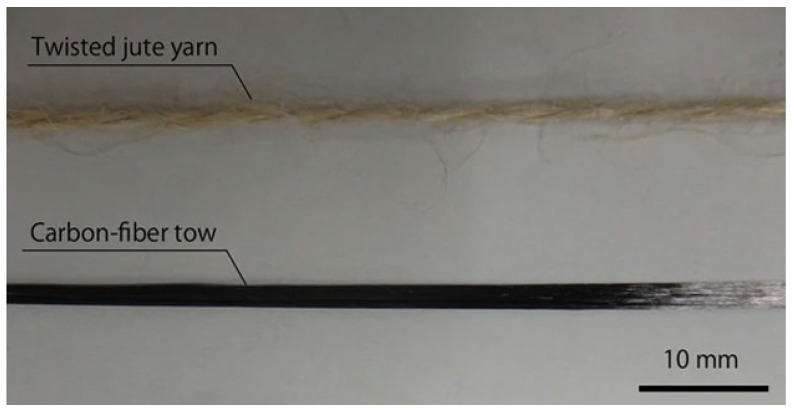
Continuous fiber reinforcements used for 3D printing ([16], reproduced under a CC BY 4.0 license).

**Figure 3:**
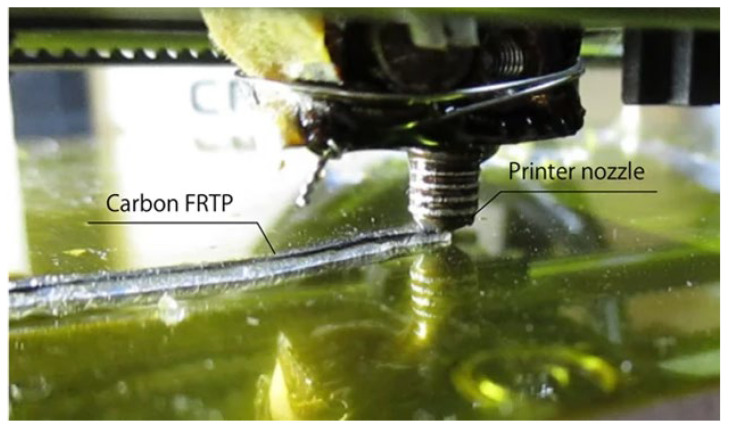
Photograph of the 3D printing of a CFRTP ([16], reproduced under a CC BY 4.0 license).

This technique allows the direct production of 3D components without the use of molds and is considered a possible future standard methodology for the fabrication of advanced composites. In the process, a thermoplastic filament and continuous fibers are supplied to the 3D printer separately. The fibers are impregnated with thermoplastic resin inside the heated nozzle just before printing. Polylactic acid (PLA) was used as the thermoplastic matrix, while reinforcement options include both carbon fibers and natural jute fibers (see Figure 2). The results obtained demonstrate that carbon fiber-reinforced composites exhibit superior mechanical properties compared to jute-reinforced composites and non-reinforced polymers. This method (Figure 3) represents a notable advancement in the production of 3D-printed composites, with significant potential for future applications.

Processes have also been developed for the recycling of components produced with continuous carbon fiber and PLA. In this innovative process, continuous carbon fibers are recycled from 3D printed continuous fiber-reinforced thermoplastic composites (CFRTPC) to produce PLA-impregnated carbon filaments. These recycled filaments perform better than the original ones. Pure PLA and recycled carbon filaments are then used to remanufacture composites with significantly improved mechanical properties. This approach reduces waste and promotes sustainability in CFRTPC production, despite a slight increase in energy consumption. Overall, it represents a significant step toward eco-friendly CFRTPC production [17].

It has also been studied how carbon fibers are particularly suitable compared to other types of fiber to obtain a significant increase in mechanical properties [18]. The study of Dickson et al., 2017 [19] evaluates the performance of composites reinforced with continuous carbon, Kevlar, and glass fibers made using the fused deposition (FDM) 3D printing technique. Fiber-reinforced nylon composites were fabricated using a Markforged “Mark One” 3D printing system. The mechanical properties of the composites were evaluated in both tension and bending. The influence of fiber orientation, fiber type, and fiber volume fraction on the mechanical properties were also examined. The results were compared with unreinforced nylon samples and with property values known from the literature. It was found that of the fibers examined, those fabricated from carbon fibers provided the greatest increase in mechanical strength per fiber volume. Their tensile strengths were up to 6.3 times higher than those obtained with the unreinforced nylon polymer.

Heidari-Rarani 2019 [20] have developed an innovative extruder tailored for 3D printers utilizing the fused deposition modeling (FDM) technology to fabricate continuous fiber-reinforced thermoplastic composites (CFRT). They tackled challenges related to fiber tension, fiber surface preparation, printing temperature, and extruder feed rate to ensure the production of high-quality composites. The resulting extruder can be easily mounted on existing FDM 3D printers without the need for a complete redesign. Experimental testing involved both pure PLA and carbon fiber-reinforced PLA specimens, showcasing significant enhancements in mechanical properties.

Other studies have investigated the behavior of continuous carbon fiber with PEEK (poly-ether-ether-ketone). For example, Luo et al., 2019 [21] have studied continuous carbon fiber-reinforced poly-ether-ether-ketone (CCF/PEEK) composites. They have been prepared using extrusion-based 3D printing, with a particular focus on addressing the issue of interlayer delamination. To overcome this challenge, the impregnation behavior between carbon fiber tows and PEEK was investigated by adjusting the matrix material’s viscosity and implementing pre-impregnation. Additionally, the interlayer bonding behavior was studied under different laser preheating temperatures. The results showed that effective impregnation and improved interlayer bonding in CCF/PEEK composites were achieved. The interlaminar shear strength and flexural strength of these composites could exceed 35 MPa and 480 MPa, respectively, making them promising candidates for lightweight, high-strength composite structures in industrial applications.

Jahangir et al., 2019 [22] used an approach to reinforce polycarbonate (PC) 3D-printed parts by inserting continuous bundles of carbon fiber (CF). They made ASTM D638 Class I pattern samples with gaps in the print to manually insert and embed the carbon fiber bundles. The samples contained one, two, or three layers of carbon fiber bundles embedded via a manual lay-up process on a fused filament fabrication (FFF) production machine. PC reinforcement, in which three bundles of carbon fiber were embedded, showed a 77% increase in tensile strength compared to molded PC without fiber. This increase in strength has been achieved thanks to the contribution of both the carbon fibers and the reduction in porosity. However, due to the difference in thermal expansion coefficients between PC and carbon fibers, the so-called warping phenomenon has occurred, whereby the components bend during the printing process caused by heat. The inclusion of carbon fibers in the molded PC samples led to a large increase in specific strength (from 2.9 MPa/g to 4.9 MPa/g) with a small increase in total mass (from 9.4 g to 9.7 g). Pull-out tests have shown that the adhesion between the carbon fiber fabrics and the PC is strong enough that the carbon fibers would break before being pulled out of the CF-PC interface.

Other studies have used innovative additive techniques to impregnate carbon fiber with the plastic material, with Li et al., 2020 [23] highlighting the benefits of microwave 3D printing technology over traditional heating methods. This technology allows for faster and more precise prints, especially for high-performance continuous carbon fiber composites. However, variations in printing speed, due to changes in vector direction during the process, pose a challenge. To address these variations, the text introduces a new temperature control method called the “prediction-model and combined step temperature control (PMSP) method”. Compared to traditional PID control, PMSP significantly reduces the standard deviation of the printing temperature, ensuring more consistent prints. Furthermore, the results demonstrate that microwave 3D printing technology can produce continuous carbon fiber composites with significantly higher tensile strength than conventional printing methods at low speeds.

Parandoush 2019 [24] introduced a novel approach inspired by layered object additive manufacturing (LOM) that has been introduced for 3D printing continuous carbon fiber-reinforced CFRTP using prepreg composite (prepreg) sheets. The prepreg sheets are shaped based on the CAD design divided into layers orthogonal to the stacking direction, and then the adjacent layers are bonded together using a CO2 laser beam and a roller system for consolidation. This innovative method has resulted in minimal void spaces and strong interlaminar bonding, leading to the highest reported tensile strength (668.3 MPa) and flexural strength (591.16 MPa) for all CFRTPs produced through additive manufacturing. Additionally, this technique enables precise control of carbon fiber alignment in different layers, allowing for CFRTPs with unidirectional fiber reinforcement, cross-layered structures, and [0/−45/0/45] reinforcements.

Compared to other 3D printing techniques, like FDM, SLA, SLS, inkjet, and extrusion, the mechanical properties of these 3D-printed CFRTP composites are superior, whether with continuous or short carbon fiber reinforcement. This methodology enables the creation of highly customized CFRTP geometries with complex designs, optimizing costs and flexibility when compared to conventional production methods. Furthermore, the technology is easily scalable for large-scale manufacturing. In the study, a finite element analysis (FE) model was developed to predict temperature distribution in the CFRTP structure during the laser-assisted 3D printing process. This model proves valuable in optimizing the technique for various structural composites. To summarize, this technology offers the capability to produce components of varying sizes, ranging from small to large, making it highly advantageous for large-scale commercial production. With its process flexibility and the ability to construct strong and lightweight structures, this technology holds significant potential across diverse industries, including aerospace, automotive, marine, and civil engineering, where automation can effectively reduce costs and waste.

Zhang et al., 2022 [25] concentrated his studies on 3D printing carbon fiber-reinforced polymer (CCFRP) composites, using an in-situ impregnation technique. A new 3D-printed extrusion technology was developed to generate these composites, achieving low voids and good mechanical properties thanks to proper impregnation of the carbon fibers. Analysis of the printing pressure during the process revealed that a thinner printing layer generates a greater ironing force, reducing void formation and improving the surface quality and mechanical properties of the printed components. However, excessively high tensile strength can compromise dimensional accuracy, especially in regions with high curvatures, due to incomplete solidification of the matrix. Therefore, it is necessary to find a balance between dimensional accuracy and mechanical properties when optimizing 3D printing parameters.

Chang et al., 2020 [26] presents a study on continuous carbon fiber-reinforced poly-ether-ether-ketone (CCF/PEEK) composites using the laser-assisted laminated object manufacturing (LA-LOM) technique. The authors explored the impact of laser power and consolidation speed on the flexural strength of the composites to identify the optimal process parameters: 55 W laser power and 40 mm/s consolidation speed. Additionally, hot press postprocessing (HPP) was applied to further enhance the mechanical properties of the samples. The findings revealed that the 3D-printed CCF/PEEK composites with HPP exhibited an impressive flexural modulus of 125.7 GPa and a robust flexural strength of 1901.1 MPa. Furthermore, the tensile modulus and strength were measured at 133.1 GPa and 1513.8 MPa, respectively. The unidirectional CCF/PEEK composites demonstrated exceptional flexural strength of 670.5 MPa and a tensile strength of 1212.9 MPa after HPP. Various fiber alignment samples were prepared and characterized. The CCF/PEEK composites showcased superior tensile performance compared to carbon fiber-reinforced thermoplastics (CFRTP) produced through fused filament fabrication (FFF), primarily due to their high carbon fiber content and strong interfacial bonding between prepreg layers.

Chen et al., 2019 [27] revolves around laser sintering (LS) of composites containing carbon fiber (Cf) combined with polymeric powders. The study provides evidence that incorporating Cf within the polymer grains, referred to as “composite grain powder” in the paper, leads to enhanced mechanical performance. The actual Cf content in the composite powders varies from 33% to 54%, different from the initial 30%. Detailed analyses were carried out on different Cf/PEK composite powders obtained through milling, as well as a dry blend of Cf/PEK with 30% Cf by weight. The composite powders exhibited superior laser sintering capabilities compared to the dry blends. Scanning electron microscope (SEM) images of laser-sintered films provided valuable insights into the distinctions between dry blends and composite powders. Dynamic mechanical thermal analysis (DMTA) tests demonstrated a significant increase in complex moduli (E*) for laser-sintered samples using composite powders, outperforming both dry blends and pure polymer HP3 PEK. The presence of Cf contributed to the improved complex modulus of the composite films. Moreover, the study identified the “Fraction B” composite powders as the optimum choice for laser sintering due to their ability to maintain desired properties and achieve superior performance when compared to laser-sintered films using other composite powders.

Klift et al., 2016 [28] evaluated the production capabilities of the “Mark One”^®^ 3D printer in fabricating carbon fiber-reinforced thermoplastic (CFRTP) tensile test specimens following the JIS K 7073 standard, using fused deposition modeling technology. Various types of CFRTP tensile test samples were manufactured and subjected to tension tests to evaluate the mechanical properties of the 3D-printed CFRTP material. The primary objective of this research was to enhance the understanding of the CFRTP 3D printing process and subsequently enhance it to yield stronger 3D-printed CFRTP materials. By utilizing the Mark One^®^ machine, they successfully achieved CFRTP specimens with continuous carbon fiber alignment. However, the presence of fiber discontinuities resulted in premature failure in areas without fibers, significantly reducing the tensile strength of the composite material.

The development of additive manufacturing techniques for continuous carbon fiber-reinforced thermoplastic composites (CFRTP) is rapidly progressing due to its exceptional flexibility in creating three-dimensional structures compared to traditional manufacturing methods. However, certain challenges, such as weak bonding between layers, void spaces between beads and layers, and low carbon fiber volume, have been identified in the literature, restricting the application of these methods in critical industries, like aerospace and defense.

The difficulties related to the integration of continuous carbon fiber into a polymer matrix have been addressed in many works. Zhuo et al., 2022 [29] studied the limitations of CF-3DP (continuous fiber 3D Printing) composites’ mechanical performance including issues like low fiber volume, incomplete impregnation, high voids, and uneven fiber distribution. The research focused on using continuous carbon/PA6 fiber bundles as feedstock and employed an automated pultrusion process. An open-source FFF 3D printer was adapted for CF-3DP. The results showed fiber volume ranging from 44% to 47% and void content between 0.5% and 2%. While CF-3DP materials exhibited strength in the fiber direction, they had lower properties in the matrix-sensitive directions. Further research is needed to improve CF-3DP composite properties.

The efforts made by research to advance the development of additive manufacturing technologies in the field of continuous carbon fiber composites are an example of how important this technology is for the evolution of production systems. Many studies have focused on the analysis of the mechanical properties of the structures obtained with the aforementioned technology [30,31,32,33,34,35]. The discussion proceeds by analyzing more specifically the mechanical properties of thermoplastic composites containing continuous carbon fiber. Melenka et al., 2016 [36] carried out a study with the aim of evaluating the elastic properties of fiber-reinforced 3D-printed structures and predicting these elastic properties using a stiffness averaging (VAS) method. In the study in question, the tensile properties of 3D-printed and fiber-reinforced components produced by the “MarkOne” 3D printer were evaluated. The specimens studied in this work were produced by varying the volume fraction of the fibers within the 3D-printed structures (4.04, 8.08, and 10.1%, respectively). The experimentally determined modulus of elasticity was found to be 1767.2, 6920.0, and 9001.2 MPa for fiber volume fractions of 4.04%, 8.08%, and 10.1%, respectively. The predicted elastic modules were found to be 4155.7, 7380.0, and 8992.1 MPa. The model results differed from the experiments by 57.5%, 6.2%, and 0.1% for the respective fiber volume fractions. The test results showed that an increase in the volume of fiber reinforcement leads to an increase in the stiffness and ultimate strength of the test specimens. Furthermore, there are numerous works that aim to correlate the mechanical properties with the production process [37,38,39], this objective is fundamental in the technological development of this technique, as the cause–effect relationship between production methodology and finished product is essential. An example of how printing parameters can impact performance is seen in the interlaminar behavior of print products. The adhesion between layers is fundamental in continuous 3D printing of carbon fiber as it represents the weak point of this technology. This adhesion has been analyzed in various works [40,41] and is believed to be related to certain printing parameters. Tian et al., 2016 [42] researched an innovative manufacturing process based on 3D printing of continuous fiber-reinforced thermoplastic composites (CFRTPC). In this printing process, continuous carbon fiber filaments and PLA were used as the matrix, respectively, they were simultaneously fed into the fused deposition additive manufacturing (FDM) process. It has been found that temperature and pressure are fundamental parameters for the printing process, determining the final mechanical properties of the composites. A systematic investigation was conducted into the influence of printing parameters on the interfaces and performance of printed composites. Complete impregnation of the thermoplastic materials in the fiber bundle was achieved when the polymer temperature was between 200 and 230 °C. The strength of the bond between the various layers could be guaranteed with a layer thickness of between 0.4 mm and 0.6 mm. With optimized process parameters, 3D-printed CFR PLA composites with a fiber content of 27% can achieve maximum flexural strength of 335 MPa and a flexural modulus of 30 GPa. It is also relevant to examine the behavior of thermoplastic and continuous fiber components in relation to impact resistance. The study of Caminero et al., 2018 [43] deals with continuous fiber-reinforced thermoplastic composites 3D printed using fused deposition modeling (FDM) technology. The text highlights the importance of FDM as a promising additive manufacturing technology for composites and the rise of continuous fiber-reinforced thermoplastic composites in industrial applications due to their excellent mechanical performance and recyclability characteristics. One of the main objectives of the study is to analyze the effect of impact damage on the structural integrity of 3D-printed composites compared to traditional prepreg composites. In particular, the build orientation, layer thickness, and volumetric content of the fibers are examined. The results indicate that build orientation and layer thickness affect impact resistance differently for flat and vertical specimens, with more brittle fracture in vertical specimens. The overall results reveal that, in some cases, 3D-printed composites significantly outperform traditional 3D-printed thermoplastic materials and can be compared to conventional prepreg materials in terms of impact resistance. To end this section, it is important to underline that continuous carbon fibers are not the only ones subjected to research and capable of obtaining excellent results in the additive production of long fiber composites. Continuous glass fibers constitute a valid alternative, from the point of view of mechanical properties, compared to continuous carbon fibers [44,45,46,47].

### 2.2. 3D-Printed Short Carbon Fiber-Reinforced Composites

Various studies have evaluated an innovative family of technologies for the production of short fiber-reinforced components [48,49,50,51,52], expanding the options available to designers. Fiber-reinforced AM composites are characterized by the fact that various process-related parameters, such as the amount of reinforcing fiber or the printing architecture, can predominantly influence the tensile properties of the final parts [53]. To optimize the production of components made using these new AM technologies [54], an extensive preparatory phase of studies is needed to lay the guidelines for the design of fiber-reinforced 3D-printed parts. It is precisely necessary to evaluate the effects that the different geometric parameters have on the tensile properties of 3D-printed composites produced using the filament fusion (FFF) technique. In this section, both the production technologies of short fiber composites using AM and the mechanical characteristics will be presented together. This choice lies in the fact that, unlike additive manufacturing which involves long fiber products, the additive manufacturing of short fiber composites does not present too many complexities regarding the architecture of the machine, as the operation is essentially that of a classic FDM machine that prints only polymer.

Ferreira et al., 2017 [55] characterized materials produced at a mechanical level through FDM additive manufacturing techniques, using PLA and PLA+CF or PLA reinforced with short carbon fibers with a percentage of 15%. The chosen approach considers materials as orthotropic layered structures with specific orientations. The mechanical properties examined are as follows: strength modulus (E1 and E2), Poisson’s ratios (n12 and n21), shear modulus (G12), as well as tensile strength (S1 and S2) and shear strength (S12). The testing was carried out on samples with unidirectional arrangements or specific orientations. The results highlight a significant increase in stiffness in the printing direction (E1) for PLA+CF, with an increase of approximately 2.2% compared to PLA. A 25% improvement in transverse strength modulus (E2) and 16% improvement in shear modulus (G12) was detected. Furthermore, the fibers contribute to diversifying the Poisson’s ratios (n12 is 2.5 times higher than n21) in PLA+CF, in line with the increase in stiffness in the printing direction. However, the tensile strength (S1 and S2) and the shear strength (S12) do not undergo significant changes due to the addition of fibers. Another aspect to consider is the length of the carbon fibers, estimated at 60 µm, which may be insufficient to make significant improvements in strength properties. Furthermore, the limited adhesion between PLA and carbon fibers appears to contribute to the greater fragility of the reinforced material. SEM analysis reveals how the short carbon fibers in the PLA+CF composite are mainly aligned with the printing direction, explaining the notable increase in stiffness in this direction.

In a study on the fabrication of thermoplastic matrix CFRP composites using the fused deposition modeling (FDM) technique, Ning et al., 2015 [56] studied the impact of the addition of carbon fibers on the mechanical properties of the samples. The main results are reported below. The addition of carbon fibers to plastic materials resulted in an increase in tensile strength and Young’s modulus; on the other hand there was a decrease in toughness, yield strength, and ductility. The samples with a percentage of 5% by weight of carbon fibers showed on average the highest tensile strength values, with an increase of 22.5%. At the same time, samples with 7.5 wt% carbon fibers showed the highest average values of Young’s modulus, with an increase of 30.5%. Samples with 150 µm length carbon fibers demonstrated higher tensile strength and Young’s modulus than those with 100 µm length carbon fibers. However, they showed lower toughness and ductility, without significant differences in yield strength. Compared to samples composed solely of thermoplastic polymers, CFRP composite samples with a 5% weight percentage of carbon fibers presented higher flexural strength, flexural modulus, and flexural toughness, with increases of 11.82%, 16.82%, and 21.86%, respectively. Furthermore, it was found that increasing the percentage of carbon fibers to 10 wt% led to an increase in porosity in the samples, resulting in a decrease in the average values of tensile strength, toughness, yield strength, and ductility.

Papon et al., 2019 [57] evaluated the fracture properties (stress intensity factor and energy release rate) of materials produced by FDM 3D printing using polylactic acid (PLA) and composites reinforced with short carbon fibers (CF). The goal was to understand how CF reinforcement, nozzle conformation, and bead orientation influenced fracture characteristics, void contents, and inter-material adhesion. During the study, samples made using the filament fusion (FFF) manufacturing technique using circular and square nozzles were compared with traditional samples manufactured using compression printing (CM). Compact tension (CT) samples with different CF concentrations (0%, 3%, 5%, 7%, and 10%) were produced with two layering orientations (45 degrees/−45 degrees and 0 degrees/90 degrees) using PLA filaments and CF/PLA composites. The results demonstrated that CF/PLA composites exhibited a significant increase in fracture toughness and higher fracture energy compared to pure PLA. At 5% CF, fracture toughness increased by 42% for the 0 degree/90 degree bead orientation and by 38% for the 45 degree/−45 degree orientation. Fracture energy increased by 77% for the 0 degree/90 degree orientation and by 88% for the 45 degree/−45 degree orientation with the same fiber concentration. In addition, samples made with square-shaped nozzles exhibited higher fracture toughness with fewer bead gaps and larger bond areas compared to circular nozzles. It was also observed that the fibers were mainly aligned along the beads produced due to the FFF process. In terms of fracture mechanism, the 0 degree/90 degree orientation saw a greater contribution from the 90 degree curbs, while the 45 degree/−45 degree orientation saw contributions from both the 45 degree and −45 degrees. However, no improvement in fracture characteristics was observed with higher fiber concentrations (7% and 10%). This was attributed to void spaces within the beads, microcracks, a weak bond between the fibers and the PLA matrix, and void spaces around the surface of the fibers. To address these issues, proper surface treatment of the fibers, use of appropriate sizing agents prior to 3D printing, and annealing to reduce internal bead void spaces and improve the interface between the fibers and the matrix are recommended. In summary, the study highlighted the important role of carbon fiber reinforcement, nozzle geometry and, bead orientation on the fracture characteristics of materials produced with 3D printing, underlining the opportunity for further research to optimize these parameters and improve the performance of the materials. It can be seen from the various studies analyzed that the study of composites of thermoplastic matrix and short carbon fibers, produced using additive manufacturing techniques, is fundamental for the evolution of this technology, as the performance of the finished products is strictly correlated with a series of parameters production characteristics characteristic of 3D printing. The parameters capable of influencing the final characteristics of this type of composites are not easy to identify and numerous works have attempted to identify and study them in order to optimize the processes [58,59,60,61].

**Figure 4:**
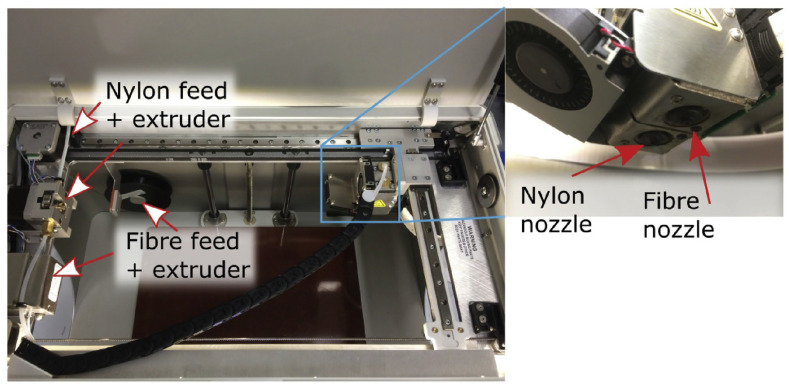
Overview of the MarkOne printer with the dual nozzle system to print nylon filament and fiber filament ([62], reproduced under a CC BY 4.0 license).

Blok et al., 2018 [62] examined 3D printing compositional blends for FFF, where carbon fibers are embedded in a thermoplastic matrix in order to increase its strength and stiffness (see Figure 4). 

**Figure 5:**
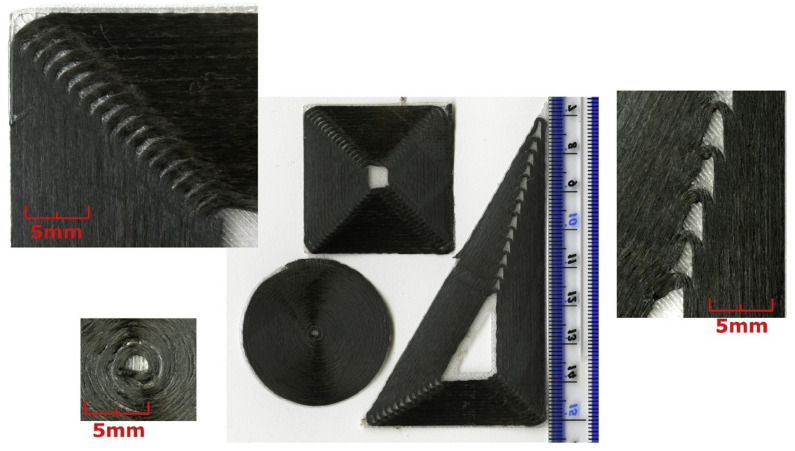
Benchmark prints for Mark One printer with detail of corner radii ([62], reproduced under a CC BY 4.0 license).

Experimental research was conducted to compare blends with short fibers and blends with continuous fibers (see Figure 5). Printing continuous carbon fibers using the “Mark One” printer was found to result in significant performance improvements over unreinforced thermoplastics, with mechanical properties of a similar magnitude to typical unidirectional epoxy matrix composites.

However, the method has limitations in design freedom, as the fragile continuous carbon fibers cannot be deposited smoothly through small bending radii and sharp corners.

Filaments with embedded carbon microfibers of short length (approximately 100 μm) demonstrate improved printing capabilities and are suitable for use with standard printing methods but offer only a modest increase in mechanical properties compared to purely thermoplastic characteristics. The hypothesis is formulated that increasing the length of the fibers in filaments with short fibers can lead to an increase in mechanical properties, potentially approaching those of composites with continuous fibers, while maintaining the high degree of freedom in the design of the process itself (FFF). The performance of two of the currently most advanced solutions was evaluated through mechanical tests. The tensile strength and stiffness of parts printed with continuous fibers were 986 MPa and 64 GPa, respectively, more than an order of magnitude higher than parts printed with short fiber-reinforced filaments (33 MPa and 1.9 GPa). A disadvantage of the continuous fiber printer, however, is limited control over the position of the fibers and the formation of voids when printing more complex shapes.

To combine the advantages of both techniques, many studies have been carried out to optimize AM processes of composite materials capable of using both continuous fiber and short carbon fiber as reinforcement. The influence on the variation in parameters, such as infill density and infill patterns for short fiber composite material, as well as fiber volume fraction and printing architecture of continuous fiber-reinforced (CFR) composites, was investigated. The influence of the position of the initial point of deposition of the reinforcing fibers on the tensile properties of the test samples was studied. Furthermore, the influence of the fiber deposition pattern on the tensile performance was quantified [63,64,65].

## 3. Comparison of Results

This paragraph presents a comparison of the different works together with the results obtained in the individual studies and the types of carbon fibers used. This comparison is provided via Table 1.

## 4. Future Developments

The challenges and prospects for carbon fiber thermoplastic composites (CFRTPCs) based on additive manufacturing (AM) technology are closely linked to current developments and emerging opportunities. Critical aspects include improving fiber adhesion, optimizing printing parameters, exploring the effect of fiber length, and addressing challenges related to interlayer interface and shear strength. Furthermore, the potential for industrial applications remains promising, thanks to the ability to create complex and lightweight structures. Continued research will help improve performance, overcome challenges, and unlock the full potential of these materials in industry and other applications.

## 5. Discussion and Conclusions

Overall, studies on thermoplastic composites reinforced with carbon fibers, both long and short, have been proven to open interesting perspectives in the field of 3D printing technology. The results clearly indicate a notable improvement in mechanical properties in CFRTPC composites. In particular, the tensile modulus increased significantly, suggesting a substantial increase in stiffness. Fiber orientation plays a determining role in mechanical performance, with increased stiffness observed in the printing direction. However, the inclusion of short fibers did not lead to highly significant improvements, as was the case with long fibers. The presence of short fibers made the material more fragile. The poor adhesion between the thermoplastic matrix and the carbon fibers is an essential area for improvement. Optimizing printing parameters is another necessity to maximize mechanical properties. Challenges remain in terms of adhesion, fiber length, and the need to optimize printing parameters. However, this technology offers considerable potential for industrial applications. The ability to create complex and lightweight structures could lead to innovative solutions in several industrial sectors.

## Figures and Tables

**Table 1:** Comparisons.

Reference	Type of Study Carried Out	Results	Type of Carbon Fibers
[36]	The elastic properties of fiber-reinforced 3D-printed structures were evaluated, and these elastic properties were predicted using a stiffness averaging (VAS) method.	Modulus of elasticity was equal to 1767.2, 6920.0, and 9001.2 MPa for fiber volume fractions of 4.04%, 8.08%, and 10.1%, respectively.	Continuous
[42]	3D printing of continuous fiber-reinforced thermoplastic composites (CFRTPC) was studied. Continuous carbon fiber and PLA filaments were used in this printing process, respectively, which were simultaneously fed into the fused deposition additive manufacturing (FDM) process.	The complete impregnation of the thermoplastic materials in the fiber bundle occurs when the polymer temperature is between 200 and 230 °C. The tightness of the bond between the various layers could be guaranteed with a layer thickness between 0.4 mm and 0.6 mm. With optimized process parameters, 3D-printed CFR PLA composites with a fiber content of 27% can achieve a maximum flexural strength of 335 MPa and a flexural modulus of 30 GPa.	Continuous
[43]	One of the main objectives of the study is to analyze the effect of impact damage on the structural integrity of 3D-printed composites compared to traditional prepreg composites. In particular, the build orientation, layer thickness, and volumetric content of the fibers are examined.	Build orientation and layer thickness affect impact resistance differently for specimens printed flat and vertically. Brittle fractures in specimens produced in vertical orientation. In some cases, the 3D-printed composites significantly outperform traditional 3D-printed thermoplastic materials and can be compared to conventional prepreg materials in terms of impact resistance.	Continuous
[15]	This study analyzes the use of 3D printing technology to produce continuous carbon fiber-reinforced polylactic acid (PLA) composites.	Tensile and flexural strengths of preprocessed carbon fiber-reinforced composites are 13.8% and 164% higher than untreated samples, respectively. The storage modulus is also higher than pure PLA and untreated samples, with increases of 166% and 351%, respectively.	Continuous
[19]	In this study, the performance of continuous carbon-reinforced composites made using the fused deposition (FDM) 3D printing technique with a nylon matrix was evaluated.	Their tensile strengths were up to 6.3 times higher than those obtained with the unreinforced nylon polymer.	Continuous
[21]	In this work, continuous carbon fiber-reinforced poly-ether-ether-ketone (CCF/PEEK) composites were studied. They were prepared using extrusion-based 3D printing, with particular attention to the issue of delamination of the intermediate layers.	The interlaminar shear and flexural strength of these composites could exceed 35 MPa and 480 MPa, respectively.	Continuous
[22]	In this work, an approach was used to strengthen polycarbonate (PC) 3D-printed parts by inserting continuous carbon fiber (CF) bundles.	Reinforced PC showed a 77% increase in tensile strength compared to PC printed without fiber.	Continuous
[24]	In this study, a new layered object additive manufacturing (LOM)-inspired approach was introduced for 3D printing continuous carbon fiber-reinforced CFRTP using prepreg composite sheets.	Minimal void spaces and strong interlaminar bonding were produced, leading to a maximum tensile strength of 668.3 MPa and flexural strength of 591.16 MPa.	Continuous
[26]	In this work, continuous carbon fiber-reinforced poly-ether-ether-ketone (CCF/PEEK) composites were studied using the laser-assisted lamination object manufacturing (LA-LOM) technique.	The 3D-printed CCF/PEEK composites with hot press postprocessing (HPP) exhibited a flexural modulus of 125.7 GPa and a robust flexural strength of 1901.1 MPa. Tensile modulus and strength were measured at 133.1 GPa and 1513.8 MPa, respectively. The unidirectional CCF/PEEK composites demonstrated exceptional flexural strength of 670.5 MPa and tensile strength of 1212.9 MPa after hot press postprocessing.	Continuous
[55]	In this study, materials produced through FDM additive manufacturing techniques were characterized at a mechanical level, using PLA and PLA+CF reinforced with short carbon fibers with a percentage of 15%.	A significant increase in stiffness in the printing direction (E1) for PLA+CF, with an increase of approximately 2.2% compared to PLA alone, was observed. A 25% improvement in transverse strength modulus (E2) and 16% improvement in shear modulus (G12) was appreciated compared to PLA alone.	Short
[56]	In this work, the impact of the addition of carbon fibers on the mechanical properties of the samples was studied.	The samples with a percentage of 5% by weight of carbon fibers showed on average the highest tensile strength values, with an increase of 22.5%. Samples with 7.5 wt% carbon fibers showed the highest average values of Young’s modulus, with an increase of 30.5%. Samples with 150 µm length carbon fibers demonstrated higher tensile strength and Young’s modulus than those with 100 µm length carbon fibers.	Short
[57]	In this study, the fracture properties (stress intensity factor and energy release rate) of materials produced by FDM 3D printing using polylactic acid (PLA) and short carbon fiber (CF)-reinforced composites were evaluated.	CF/PLA composites exhibited a significant increase in fracture toughness and higher fracture energy compared to pure PLA. At 5% CF, fracture toughness increased by 42% for the 0 degree/90 degree bead orientation and by 38% for the 45 degree/−45 degree orientation. Fracture energy increased by 77% for the 0 degree/90 degree orientation and by 88% for the 45 degree/−45 degree orientation with the same fiber concentration.	Short
[62]	In this work, 3D printing compositional blends for FFF were examined, in which carbon fibers are embedded in a thermoplastic matrix to increase its strength and stiffness.	The tensile strength and stiffness of parts printed with continuous fibers were 986 MPa and 64 GPa, respectively, more than an order of magnitude higher than parts printed with short fiber-reinforced filaments (33 MPa and 1.9 GPa).	Short–continuous
